# Mapping the existence and content of heading coaching documents across FIFA members associations

**DOI:** 10.1016/j.jsampl.2026.100149

**Published:** 2026-07-15

**Authors:** William Wright, Aimie Peek, Giulia Sesa, Ringo Teahan, Kerry Peek

**Affiliations:** a Discipline of Physiotherapy, Sydney School of Health Sciences, Faculty of Medicine and Health, The University of Sydney, Sydney, Australia; b John Walsh Centre for Rehabilitation Research, Kolling Institute, Northern Sydney Local Health District, Sydney, Australia; c Department of Public Health and Primary Care, Centre for Biomedical Ethics and Law, KU Leuven, Belgium; d Department of Sport and Social Sciences, Norwegian School of Sport Sciences, Norway; e FIFA Medical, Fédération Internationale de Football Association, Zurich, Switzerland

**Keywords:** Coaching, Curriculum, Head injuries, Primary prevention, Soccer, Sports medicine

## Abstract

**Background:**

Emerging evidence suggests head injury risk in football may be reduced through improved heading technique, yet it remains unclear whether heading is included within national coaching curricula or other endorsed coaching documents. This study investigated the existence, and content of heading coaching curricula/documents across FIFA Member Associations (MA).

**Methods:**

A qualitative document analysis was performed. Pertinent documents were collected by: 1) a survey completed by representatives from each MA outlining the existence of any coaching documents that included heading, 2) a review of each MA website, and 3) a web-browser search. Data were analysed using conventional content analysis.

**Results:**

From 211 MAs, 71 unique survey responses were received. Thirteen MAs (18.3%) reported having heading coaching material, of which seven provided relevant documents. Website review and browser searches identified six additional documents, yielding 13 documents with specific heading guidance. Six common categories were identified within coaching documents 1) age at which heading training is introduced, 2) use of soft/light balls during heading training, 3) heading technique tips/instruction, 4) neck exercises, 5) heading drills, and 6) coordination of body in space. However, substantial differences existed in level of detail and instruction provided.

**Conclusions:**

The public availability of MAs coaching documents is limited. Where heading coaching material exists, it lacks sufficient depth for clubs to integrate independently. An important gap exists for a standardised approach to heading skill development that is co-created with coaches and players.

**Key points**

• Head injury risk in football may be reduced through improved heading technique; however, it remains unclear whether heading is systematically taught or included within national coaching curricula or other related coaching documents.
• Among 211 FIFA-affiliated member associations (MA), 13 (18.3%) reported having heading coaching material, with seven providing relevant documents. A further 16 MAs indicated plans to develop heading guidelines in the future, suggesting growing interest in this area. Six additional documents were found via the website and browser searches.
• Substantial variation was observed in the level of detail and instruction across documents. The most commonly identified component was the use of soft or lightweight balls during heading practice, with many MAs recommending a gradual progression in ball size and mass to support player development and safety.
• By examining both the prevalence and content of heading coaching curricula and documents, this study provides an important first step in describing current practices and contributing to discussions around the future standardisation of heading skill development.

## 1 Introduction

In football, heading is defined as “*performing a deliberate movement to redirect the trajectory of a ball using the head. Heading will result in either a header (with head-to-ball contact) or an attempted header (without head-to-ball contact). Heading can be either contested (duel) or uncontested (no duel)*” [1].

In recent years, the regulation of heading in football has received increasing attention from some FIFA affiliated Member Associations (MAs). While FIFA is the sport’s governing authority responsible for developing and promoting football worldwide, the 211 Member Associations (MA) are responsible for governing football locally. This includes developing specific coaching curricula and talent pathways that focus on skill acquisition and proficiency. Specifically, where this relates to heading, while some MAs have introduced restrictions or bans for specific age groups, like the United States Soccer Federation, others, like the German Football Association, have prioritised the development of heading technique through structured coaching practices and formalised coaching curricula. This diversity of approaches reflects an ongoing debate concerning the balance between potential brain health risks, particularly the inconclusive evidence linking header exposure to long-term neurological outcomes, and the recognition of heading as a fundamental technical skill that can influence both team performance and match outcomes [2]. At the same time, appropriate heading training has been suggested as a potentially protective factor against head injuries during contested headers, enabling players to execute the skill with a reduced risk of player-to-head impact [1].

Players reportedly head the ball between 1 and 9 times per match, with frequency increasing with age, with youth players performing fewer headers than adult players [3,4]. Variation in header incidence is also reflected in positional demands, with central out-field players often performing more headers than goalkeepers or wingers [5]. Across professional and youth matches, players collectively perform 70–110 headers per match, with a mean of 3–5 headers per player per match [6, 7], with headed goals contributing 18–22% of all goals in recent FIFA World Cups [8].

Heading is a complex skill, requiring precise timing, body positioning and coordination to redirect the ball with the appropriate velocity and accuracy. When a ball is played in the air, a player must evaluate their spatial position relative to the ball, opponent, and/or teammate. This requires the player to track the ball's trajectory and velocity to position themselves effectively in front of the ball and their opponent. The movement sequence to perform a standing header often begins with trunk hyperextension, followed by hip and then trunk flexion, with greater trunk flexion reportedly producing higher impact forces [3]. In jumping headers, this sequence is executed during a vertical jump timed so that generally the peak height of the jump coincides with the header [9]. Furthermore, neck strength and neuromuscular control are also critical in controlling head acceleration upon ball-to-head contact [[10], [11], [12], [13], [14], [15], [16]].

While heading coaching curricula should improve heading technique proficiency that may provide a competitive advantage to teams, there is emerging evidence to suggest that head injury risk may be reduced by improved heading technique [17]. Heading technique proficiency is the ability to consistently perform headers using optimal body movements, timing, positioning, and control to achieve a desired outcome (such as a clearance, pass, or shot on goal) while also minimising injury risk [1,18]. Multiple studies [[19], [20], [21]] have reported that youth and professional players are more likely to receive a head or brain injury such as contusions, lacerations and concussions from player-to-player contact when two or more players are competing for an aerial ball. Concussions contribute around 4–22% of all football injuries, occurring at a rate of 0.5–1.7 per 1000 playing hours [22], and more likely when a player is unaware of the oncoming contact [23]. Increased ball tracking through vision training may be effective in enhancing anticipation and reducing concussion in sports [24].

Furthermore, research suggests that teaching players effective and protective body positioning may reduce the risk of head impacts events [1]. Effective and protective body positioning in heading refers to how a player positions their body before and during a header to maximise both performance (power, accuracy) and minimise injury risk (which may include head positioning to increase neck muscle activation to reduce head acceleration during the header, as well as the use of their upper body to protect their head from an opponent’s elbow during a contested aerial duel) [1]. With limited evidence that neck muscle engagement during a header may be improved through the inclusion of neuromuscular neck training in injury prevention programs [[2], [25], [26]].

However, there is currently a paucity of evidence on how heading is taught within MAs. For example, it has been reported that approximately 50% of amateur players in Australia have never received heading specific training [27,28]. Given the complexity associated with developing skills in heading, the importance of headers in both attacking and defensive play, and the potential for skills-based training to reduce head injury risk it would appear logical that heading technique is included in MA coaching curricula. This study aimed to investigate the existence, and content of heading coaching curricula across FIFA affiliated MAs.

## 2 Methods

### 2.1 Study design and ethical considerations

A qualitative document analysis, defined as a systematic method for generating context and insights from policy documents [29], was conducted. The study employed the ‘READ’ approach [30], ready materials, extract data, analyse data, and distil findings, as a structured technique for document collection, data extraction, and analysis. Within each step of the READ approach, procedures were informed by the approach used in an earlier study during their examination of MA return-to-play guidance documents [31].

Data were collected between December 2024 and June 2025. As this study did not involve the collection of personal or patient-related data, it was exempt from formal ethics approval by the University of Sydney. Nonetheless, the study followed the National Statement on Ethical Conduct in Human Research [32], ensuring adherence to the core principles of respect, integrity, and merit.

1. Readying heading coaching material – Data preparation

To improve the comprehensiveness of the search, three data collection methods were used: 1) representatives from each MA were invited to complete a survey to outline the existence of any coaching documents within their MA that included heading, 2) a review of each MA website, and 3) a web-browser (Google, US) search. Coaching curricula or related documents refer to any structured material that outlines what skills coaches should teach to players, as well as information on when and how they should teach these skills. The inclusion/exclusion criteria for coaching documents are outlined in Table 1.

**Table 1:** Inclusion/exclusion criteria.

Inclusion criteria	Exclusion criteria
Published by a recognised FIFA Member Association	Not authored or endorsed by the Member Association
Explicit coaching material (curriculum, course, or guidelines)	General news, media releases, or opinion articles without coaching content
Available as a digital copy	Duplicate versions of the same curriculum (only latest retained)
To be included in the analysis: Must contain reference to headers or heading	Inaccessible due to paywalls, broken links, or restricted access

### 2.2 Survey

Medical and coaching representatives from each MA within FIFA’s database were contacted via email with an invitation to complete an online survey (Qualtrics, Provo, UT), with two reminder emails sent 4 weeks apart. Respondents were asked to answer the following questions:

1. Please tell us the name of the member association you represent.
2. To which confederation is your association affiliated?
3. What is your role within this association?
4. Does your association have a heading in football coaching curricula? (yes/no/unsure)
5. If yes, please attach a PDF copy here or email a copy to medical@fifa.org
6. If no, does your association have in development or plan to develop a heading in football coaching curricula?

For survey respondents who reported having a heading coaching curriculum but did not attach documentation, follow-up emails were sent on an ad-hoc fortnightly basis. Any curricula provided through this follow-up were incorporated into the dataset.

### 2.3 Member association website check

A comprehensive review of all MAs official websites was undertaken. This process involved a manual, page by page audit of each site, systematically navigating publicly accessible tables, dropdown menus and subpages, with a focus on sections labelled: “*Coaching*,” “*Education*,” “*Development*,” “*Resources*,” “*Regulations*” and equivalents in the relevant language(s). Where sites contained a search function, targeted keyword searches were used for: Heading, Header, Concussion, Coaching, Curriculum, Coach education.

For non-English websites, searches were conducted in the website’s primary language, with website pages and necessary translated generated via Google Translate for documentation and cross checking. Each potentially relevant document (e.g. coach education course information, licensing requirements, course outlines) were reviewed. A structured extraction template was maintained in Microsoft Excel (Microsoft, US) by the first author (WW), recording for each MA: Website URL, presence/absence of heading-specific content, document title(s) and publication year(s), content type (e.g., standalone heading curriculum, embedded in broader course, safety guidelines), original language, download date and file path reference, notes.

### 2.4 Supplementary web-browser search

An additional targeted web-browser search was conducted to capture official MA coaching documents that might be located via a Google. The web-browser search strategy involved combining the name of each MA with the specific keywords “*heading,* *or* *coaching,* *or* *curriculum*”. Searches were initially conducted in English and, where relevant, subsequently repeated in the appropriate local language (e.g., *Federación Venezolana de Fútbol* + *currículo de entrenamiento de cabeceo* for Venezuela). With data extraction and analysis following the same process as those identified from the website review, Table 2.

**Table 2:** List of Member Associations with football coaching curricula.

Member Association	Confederation	Document Type	Source	Public?	Language	Heading Mentioned?
Football Australia	AFC	Curriculum	Website check	✓	English	✓
England Football	UEFA	Guideline + Curriculum	Website check	✓	English	✓
Eswatini Football Association	CAF	Curriculum	Survey	–	English	✓
Finnish Football Association	UEFA	Guideline	Survey	–	Finnish	✓
German Football Association	UEFA	Guideline	Survey	–	English	✓
Football Association of Hong Kong, China	AFC	Curriculum	Website check	✓	English	–
Football Association of Ireland	UEFA	Curriculum	Web-browser search	✓	English	–
Japan Football Association	AFC	Guideline + Curriculum	Survey	–	English + Japanese	✓
Malta Football Association	UEFA	Guideline	Survey	–	English	✓
Montserrat Football Association	UEFA	Guideline	Survey	✓	English	✓
New Zealand Football	OFC	Curriculum	Web-browser search	✓	English	–
Norwegian Football Association	UEFA	Guideline + Curriculum	Survey + Web-browser search	✓	Norwegian + English	✓
Philippine Football Federation	AFC	Curriculum	Web-browser search	✓	English	–
Scottish Football Association	UEFA	Guideline	Survey + Website check	✓	English	✓
Football Association of Singapore	AFC	Curriculum	Web-browser search	✓	English	✓
South African Football Association	CAF	Curriculum	Web-browser search	✓	English	–
US Soccer	CONCACAF	Guideline + Curriculum	Survey + Web-browser search	✓	English	✓
Uruguayan Football Association	CONMEBOL	Curriculum	Web-browser search	✓	Spanish	–
Football Association of Wales	UEFA	Guideline + Curriculum	Survey + Website check	✓	English	✓

Key: AFC (Asian Football Confederation), CAF (Confederation of African Football).

CONCACAF (Confederation of North, Central, and Caribbean Association Football), CONMEBOL (Confederation of South American Football), OFC (Oceania Football Confederation), UEFA (Union of European Football Associations).

Data from the survey, website review, and web-browser search were compiled, and independently checked by the first author (WW) with oversight from the research team. Duplicate responses were also reviewed, for example, identical responses from the same representative where the duplicate was deleted. Where multiple representatives from one MA submitted surveys, these were combined. Partial or incomplete responses were also preserved to indicate survey engagement.

2. Extracting data

Dalglish et al. [30]; describe document analyses as “*first and foremost exercises in close reading*.” This involved reading and re-reading heading coaching content over a two-week period, with iterative re-readings during the development of the results section. Initially documents meeting the eligibility criteria (Table 1) were searched for the keyword “*head*”. Preliminary notes and emergent concepts were then recorded in an Excel spreadsheet (Microsoft, US) alongside the verbatim data.

3. Analysing data

Data analysis followed the recommendation to apply “*specific analysis methodologies*” as part of a READ approach [30]. Accordingly, drawing on the approach used by an earlier study [31], this study employed conventional content analysis [33], combining quantitative elements and qualitative interpretation. In analysing the content of coaching documents, emergent codes were established inductively by the first author (WW) through multiple line-by-line reviews of the included documents [34]. Codes were restricted to semantic meaning and interpretatively summarised to create a succinct, shorthand descriptions of the coaching information. Categories emerged when patterns of code recurred across MA material. All identified categories were reviewed to develop a coherent understanding of MA coaching material, with each category reviewed, evaluating each category’s clarity, boundaries, and representativeness by the research team. During this process of group validation, categories were kept, combined, or edited to ensure clarity and consistency in the findings.

4. Distil findings

Each proposed category was examined in relation to the research question and critiqued for transparency and internal consistency [35]. All illustrative extracts from each MA were identified to assess coherence and provide evidence of coaching content. Categories were then detailed to highlight commonalities and differences in the coaching documents.

## 3 Results

*Survey responses:* From 211 MAs, 91 responses were received, with 20 MAs responding twice, resulting in responses from 71 different MAs. Of these, 13 respondents (18.3%) reported having heading coaching material, 30 (42.3%) indicated they did not and 28 (39.4%) were unsure. Among the 13 respondents reporting material, seven provided coaching documents while six did not despite two follow up emails. Among the 30 respondents without material, 16 indicated plans to develop a heading curriculum in the future. The full survey results are located in Supplementary appendices.

*Member Association website review:* Twenty-three MA websites did not have functioning websites and/or were blocked by firewalls, resulting in a total of 188 (89.1%) MA websites inspected. Five MAs (2.7%) were identified as having coaching material through the website review.

Web-browser *search:* Six MAs were identified as having coaching materials through the web-browser search.

Across these three data collection methods, 19 MAs were identified as having publicly available, player coaching material. Details including Confederation, data source, accessibility, original language and the mention of heading are presented in Table 2. The documents analysed from each MA can be found within Supplementary Appendices.

Of the 19 MAs with specific coaching material, 13 included some content associated with heading and were included for document analysis. MAs within UEFA were the most represented (7, 53.8%), followed by MAs within AFC (3, 23.1%), followed by CONCACAF (2, 15.4%) and CAF (, 7.7%). No publicly available, heading-specific coaching materials were identified from MA's affiliated with CONMEBOL or OFC.

Table 3 summarises the content of these coaching documents categorised under different sub-headings representing the main categories that emerged from the content analysis.

**Table 3:** Content of Member Associations heading coaching material by category.

Member Association	Age of heading training introduction	Use of soft/light balls in heading training	Header technique	Neck exercises	Heading drills	Coordination of body in space
Japan Football Association	U9 (Soft balls at younger ages)	✓	✓	✓	✓	✓
Eswatini Football Association	U13	–	✓	–	–	–
Malta Football Association	U12	✓	–	✓	✓	–
Scottish Football Association	U13	✓	–	–	✓	–
Finnish Football Association	U11	–	–	✓	–	–
Germany Football Association	U7 (age appropriate ball)	✓	✓	✓	✓	–
England Football Association	U12	✓	✓	–	–	–
Montserrat Football Association	–	–	✓	–	–	–
Football Association of Wales	U12	✓	✓	–	–	–
Football Australia	U14 (Light balls if introduced earlier)	✓	–	–	–	–
Football Association of Singapore	U12	✓	–	–	–	–
Norwegian Football Association	U11 (Light balls at younger ages)	✓	–	–	–	–
US Soccer	U12	–	–	–		–

Key: ✓ content was identified; - no content was identified during document analysis.

### 3.1 Age of heading training introduction

All MAs except for Montserrat included guidance on the age that heading training should be introduced. For the majority of MAs, formal guidance occurred around the ages of U12–U14. Earlier exposure was noted in 4 MAs (Japan, Australia and Norway, Germany), using lightweight or age-appropriate balls.

### 3.2 Use of soft/light balls in heading training

Nine MAs recommend the use of soft or lightweight balls during heading practice, particularly for younger playing age groups. Most recommend a gradual progression in ball size and mass to align with player development and safety. Some recommend the use of alternative balls such as balloon or foam balls during introductory stages of heading coaching. Table 4.

**Table 4:** Summary of content related to the ball within Member Association.

Member Association	Use of soft/light balls in heading training
Japan Football Association	Balloons can be used to catch and contact the player’s forehead at a young age. Light rubber balls can also be used to practice head contact. Advice to consider ball mass and ball construction material to reduce fear, starting with very light balls (80–230 g) and progressing to sponge or pendulum balls when developing ball tracking, timing, and basic header technique.
Malta Football Association	Advice that coaches should use appropriate ball size per FIFA specifications. For header training, balls should have the lowest pressure authorised with foam balls recommended when first introducing headers.
Scottish Football Association	**U12**–**13** lightweight balls during header training. Advice to not over inflate balls and use the lowest pressure authorised by the Laws of the Game. **U16**–**17** lightweight balls are recommended to support the development of header technique.
German Football Association	**U7–U11**: Playful introduction to headers recommended using soft balls, balloons or similar. Recommended use of ultra-light balls (size 3/290 g/10.2 oz) for competitions. **U11**–**13**: Standard size 4 (350 g/12.3 oz); ultra-light balls (290 g) to train headers. **U15**: Standard size 5 (450 g/15.9 oz); use lighter balls (350 g) to practise headers, while also considering player’s different stage of football skill development.
England Football Association	Light balls to be used in header practice. Recommended to use lighter or sponge balls in header practice.
Football Association of Wales	Light balls to be used in header practice. Ball pressure should be always monitored (8.5–16psi).
Football Australia	**U13 or younger:** Start header practice using super light balls, otherwise start at U14.
Football Association of Singapore	Softer balls can also be used in header practice.
Norwegian Football Association	**U6–9:** use lightweight size 4 balls (290–320 g). **U10**–**14:** use slightly heavier balls (350–390 g), lighter and smaller than adult footballs.

### 3.3 Header technique tips/instruction

Six MAs provided guidance on header technique with a consistent focus on using the forehead, maintaining eye contact with the ball and engaging the upper body and neck when redirecting the ball. Table 5.

**Table 5:** Summary of content related to header technique tips/instruction within Member Association.

Member Association	Header technique tips/instruction
Japan Football Association	Make contact with the ball using forehead. Keep eye contact with the ball until the moment of contact. Use upper body to head the ball far away. Stabilize body to give the ball power to drive it further.
Montserrat Football Association	Hit the centre of the ball with the forehead. Keep eyes on the ball. Use entire upper body to push the ball. Keep neck strong and stable.
Football Association of Wales	Head the ball with forehead. Eyes on the ball. Keep mouth closed.
Eswatini Football Association	Contact surface: Forehead. Keep eyes open and mouth closed. Lock the neck muscles in preparation for ball contact.
German Football Association	Teaching of right header technique (frontal/forehead contact). Tension in neck muscles and whole body on ball contact.
England Football Association	Judge and adjust body position in response to the flight of the ball. Attack ball at the optimum point of the jump. Use head contact to control direction and distance of ball.

### 3.4 Neck exercises

Four MAs include specific neck muscle training exercises in their heading coaching material. Approaches ranged from basic isometric and stretching exercises to more structured programs with defined sets, repetitions and movement types. Table 6.

**Table 6:** Summary of content related to neck exercises within Member Association heading documents.

Member Association	Content related to neck exercises
Malta Football Association	In addition to the isometric neck exercises, Malta recommend training neck flexion and extension, with complementary diagrams and instructions on ‘Execution’ and ‘Starting Position’. Dosage: 20 repetitions, 2 sets.
Japan Football Association	Guidelines that introduce exercises to boost basic physical strength, such as core stability and neck strength. Neck isometric exercises and stretching exercises are included
German Football Association	Includes 3 neck exercises focusing on neuromuscular control: static flexion, static rotation, and dynamic rotation with two progression levels. Dosage: 1 set of 30 s; progression allowed.
Finnish Football Association	Incorporates 9 different neck strengthening exercises, ranging from simple age-based isometric exercises to more sport-specific variations. Dosage: 5–15 repetitions, 1–3 sets depending on endurance level.

### 3.5 Heading drills

Four MAs detail specific heading drills emphasising progressive skill development, low repetition limits and the use of thrown ball delivery methods. Most recommend unopposed short-distance header practice within younger age groups, advancing to controlled, contested headers around the ages of U16 and above. Table 7.

**Table 7:** Summary of content related to heading drills within Member Association.

Member Association	Heading drills
Japan Football Association	**U6:** Throw balloons in the air and catch; practice contacting balloon with different body parts such as forehead, foot, knee and chest. **U7/8:** Use balloon/light ball to contact with forehead; catch underhand/overhead; move and bounce [juggle] 10x on forehead. **U9/10:** Use light soft ball; bounce and head 10x; catch and head ball using forehead; bounce and try to head. **U11/12:** Use tennis ball/Size 4 ball; bounce and head 10x; jump and head changing height; play forehead catch. **U13**–**15:** Light/Size 4–5 ball; throw and catch at highest point; head 10x; jump head ball thrown from below or with a partner.
Scottish Football Association	**U12:** Max 5 headers/session, self-serve or short serves, always uncontested (i.e. not within a duel). **U13:** Balls thrown from short distances, no duel or opponent contact/interference. **U14/15 + older:** same guidance but increasing limit to 10x headers per session.
German Football Association	Gradual, low volume of headers with sufficient time between sessions. **U11/13:** Finishing drills, balls thrown not kicked deliveries for headers, no aerial duels. **U15:** Handball-heading, football tennis, balls thrown, no aerial duels. **U17/U19:** Heading-specific, crosses, technical/tactical drills. **Adults:** Thrown passes to reduce load; focus on technique for both contested/uncontested headers; introduce contested headers later; light balls can still be used.
England Football	**U12/13:** 1 session/week, light balls, max 5 headers, self-serve or short distance, unopposed. **U14**–**16:** Max 1 session/week, max 10 headers/player, varied distances. **U18:** Variety of heading situations including contested headers, introduce position specific movements. **Adult:** Max 10 headers per session, one session per week

### 3.6 Coordination of body in space

Japan Football Association was the only MA to include explicit coordination exercises aimed at developing a player’s awareness of their body in space, which included the following information:

**U6:** Catching and playing with a ball in the air or placing a balloon or newspaper to the forehead and manipulating the ball in the air.

**U7–U8:** continue these skills with game-based scenarios; examples: bounce a balloon on your forehead, throw a light ball and catch it underhanded or above your head, use a pendulum ball, move around while bouncing the ball, or join passing drills with others.

**U9+:** aerial duels, catching balls, tracking ball flight and trajectory; example: jump and compete for the ball in small-sided games, Size 4 balls in a ball net [head tennis].

## 4 Discussion

This study aimed to determine the existence, and content of heading coaching curricula or documents across FIFA-affiliated MAs. Using 3 different methods of data collection only 13 of the 211 MAs were identified as having available heading coaching material with content varying substantially in scope and detail. Collectively, these findings indicate that formal, accessible heading coaching material is limited and inconsistently implemented across MAs.

The small number of both supplied and publicly available heading coaching documents identified through the survey, website check and web-browser search may indicate that heading is not widely prioritised or systematically taught within most MAs. A finding observed in two studies of football players and coaches in Australia [[28], [36]], where approximately 50% of players and coaches reported being formally trained in heading technique. An online survey completed by 290 players, 54 coaches, 34 non-coaching staff, and 14 medical staff reported that only 45% of female players and 60% of male players had ever received formal coaching on heading, with ‘teaching heading technique based on player age, position, and experience’ being the most popular heading injury risk mitigation strategy across all cohorts [36]. Given the importance of headers in both scoring and blocking attempts at goals, the lack of heading coaching represents a gap in skill development [18].

While it is likely that additional MA coaching material exists, possibly within MA portals that limit online access to the general public, and within MA coaching courses based on confederation license programs, such as the UEFA A/B/C license, often these courses have a registration and attendance fee, and as such may not be accessible to the coaches and players who may benefit the most. For instance, most players will start to head the ball from aged 10–12 years onwards, where their exposure might be limited to coaches with limited coaching experience or training (such as a parent volunteer). Meaning that many of these players will commence performing headers in match scenarios without appropriate training in header technique within a controlled environment at training [[13], [14], [15], [16], [37]].

The limited coaching documents on heading may also reflect an incomplete understanding of how to effectively teach heading within a structured curricula or framework, as well as a lack of research on heading technique and performance [18]. While other aspects of football such as attacking, defending, passing, shooting were discussed in a wide array of MA coaching material located as part of this study, the absence of guidance on heading suggests a lack of clarity on how and when heading should be taught. Given that 16 of the 33 MAs without heading documents indicated an interest in developing future heading curricula, this likely reflects a growing global recognition of the need for heading coaching resources.

The most common recommendation was on the use of light or modified balls during heading practice, particularly in younger age groups. MAs such as Japan, Germany and Norway provided detailed, age specific guidance that progressively increased ball size and mass in line with player development and ball specifications within the Laws of the Game. Similarly, England, Wales, Australia, Singapore and Malta all advised using softer or lower-pressure balls for header training. The use of lighter balls aligns with a research that found that decreased mass or pressure can reduce head acceleration upon impact [38,[13], [14], [15], [16]] However, the effect of different size balls in reducing head impact magnitude may be negated if the ball is of a comparable pressure/inflated mass [39]. Furthermore, despite multiple MAs recommending ball modifications, there remains a lack of consistency in the level of detail provided. Some MAs refer to specific ball parameters, whilst others refer to the use of “*light*” or “*soft balls*”, requiring the interpretation of coaches and players as to the correct inflation, pressure, and mass of these alternative balls. Japan and Malta both detailed a pedagogical foundation for the introduction of header skill development using different balls, which could be used to inform more standardised heading coaching frameworks.

Few MAs provided detailed guidance on header technique. A “*frontal hit*,” or use of the forehead to strike the ball, as mentioned by the majority of those with header technique advice, aligns with research that players who head the ball with the front of their head experience lower rotational velocities compared to when the ball contacts other parts of the head [40]. Several MAs (Japan, Montserrat, Eswatini and Germany) highlighted the importance of coordinated contraction of trunk and neck muscles during a header. Similar to the effects of neck exercises, coordinated muscle activation can reduce peak head acceleration upon impact and improve ball control trajectory [41]. While instructions such as “*keep your mouth closed*” (Wales, Eswatini) may indirectly promote neck muscle activation through teeth clenching, more explicit instruction and practice may be required to maximise the force producing capacity of the neck flexors on head-to-ball contact [2]. Only Japan, Germany and England included multi-component advice of eye tracking and whole-body stabilisation during headers, therefore, an opportunity exists for more standardised guidance on header technique, potentially integrated into a broader heading coaching curriculum.

Four MAs incorporate neck muscle training such as strengthening or neuromuscular exercises into their coaching documents. While limited research [5,42] has indicated that higher neck strength is associated with lower head acceleration during headers, advice regarding which specific neck exercises should be incorporated remains unclear [2]. With unclear evidence to support the effects of neck training on reducing sports-related concussion [43]. For example, evidence suggests that isometric neck muscle training alone does not appear to reduce head acceleration upon head-to-ball contact [44,45], whereas, more promising results have been observed with neck exercises that focus on neuromuscular control, such as the exercises included within Germany’s coaching document [2]. Furthermore, the four MAs applied generalised dosages across age groups, which may compromise the effectiveness of these exercises in reducing head impact magnitude, particularly for female football players who may have lower maximal isometric neck strength when compared with male players [46]. Therefore, the limited and varied inclusion and dosage of neck training is likely reflective of this research uncertainty.

There was considerable variation in the type and intensity of heading drills recommended by MAs with heading-related material. Most MAs specified a maximum number of headers per session (ranging from 5 to 10) and typically emphasised unopposed, short distance ball delivery methods. Whilst restricting the number of headers per week adheres to the precautionary principle, there is limited evidence to support a *‘safe’* number of headers per week [2]. The implementation of short distance ball deliveries to practice header technique is supported to reduce the incidence of high impact headers [[40], [47]], which can be successfully integrated into small-sided games to enhance technical skill execution [48]. However, the benefit of this advice is limited given the lack of detail on what distances, repetitions and progressions, within the MA documents, with Japan the only MA to detail explicit coaching instructions. This lack of detail likely reflects the limited evidence to support *‘best practice*’ coaching of heading. Despite limited research supporting a pedagogical foundation for heading instruction, a recent study sed a novel programme *HeaderPrep*, to improve younger players' confidence and heading skills, as a preconditioning program before players start heading coaching in training [28]. The programme incorporated drills such as side-to-side catching, throwing, and jumping—similar to Japan’s curricula—demonstrating that structured heading drills can potentially enhance skill development.

There was a distinct lack of detail regarding the coaching of a player’s coordination of their body in space when performing a header, particularly when competing for an aerial duel. Body coordination has been highlighted as a critical skill when safely jumping and orienting the head to purposefully redirect a ball [49]. Moreover, scanning and visual awareness have been identified as important skills for header performance, could aid in reducing head injury risk, though further research is needed [1,50].

### 4.1 Limitations

Several limitations should be acknowledged. Firstly, this study only analysed self-reported survey responses and publicly available documents within MAs. Reliance on self-reported responses may have introduced response or availability bias, as document inclusion was partly dependent on the willingness and ability of respondents to share relevant materials. To mitigate this risk, multiple data collection strategies were triangulated, including conducting website checks and web-browser searches alongside the survey. Nonetheless, this approach may still not have fully captured internal or unpublished curricula or other documents within some MAs, and the researchers cannot ensure that all coaching material was identified. This limitation also does not account for the continued development and publishing of heading coaching documents in the future. Language barriers also likely limited the inclusion and interpretation of non-English documents. For example, no documents were found on the official sites of Brazil, France, or Spain. Whilst Google translate was used to translate search terms and relevant documents, translation inaccuracies may have limited the accurate interpretation or inclusion of some non-English materials. Thirdly, restricted website access due to firewalls and poor digital interfaces limited our capacity to access content, particularly those MAs with less developed online platforms. Finally, MA documents were coded solely by the first author, which may introduce subjectivity in document interpretation and coding decisions; however, coding was guided by a predefined framework to enhance consistency and transparency and reviewed as an author group.

### 4.2 Implications for future research and practice

Future research is required to examine the strategies that could be incorporated into coaching frameworks to support heading skill development, while also acknowledging the potential long-term risk associated with repeated headers across a player’s football career. Whilst this study presented the common content of MA heading coaching material, further investigation is required to determine the benefit and feasibility of implementing these strategies within practical football training contexts. To optimise relevance and applicability, such research and coaching materials should be co-designed by health professionals, researchers, coaches, and players. Accordingly, the development of future heading coaching curricula should be accompanied by a rigorously designed implementation and evaluation plan to assess the effectiveness in enhancing player proficiency and mitigating head-to-ball impact risk.

## 5 Conclusion

Heading is an important, technical part of football. Despite its prevalence, there is a paucity of evidence supporting how and when this skill should be taught. Furthermore, where such content exists, the documents often lack sufficient detail to guide coaches and players in practice. Future research and development should prioritise a standardised heading coaching curriculum, that is co-created and disseminated to support player development globally.

## Data availability

All data are included in the paper (and Supplementary appendices).

## CRediT statement

WW: methodology, validation, formal analysis, investigation, visualization, data curation, project administration, writing – original draft.

KP: conceptualisation, methodology, validation, formal analysis, resources, supervision, project administration, writing - review & editing,

AP: validation, formal analysis, resources, supervision, writing - review & editing,

GS: methodology, validation, resources, writing - review & editing,

RT: methodology, writing - review & editing.

## Ethics statement

As this study did not involve the collection of personal or patient-related data, it was exempt from formal ethics approval by the University of Sydney.

## Funding

Giulia Sesa is funded by DAiSI. This project DAiSI has received funding from the European Union’s Horizon Europe by the granting Authority REA (the European Research Executive Agency) under Grant Agreement No. 101120342; from the Swiss State Secretariat for Education, Research and Innovation (SERI) and from the Engineering and Physical Sciences Research Council (grant number: EP/Y031687/1). Funded by the European Union.

Views and opinions expressed are however those of the author(s) only and do not necessarily reflect those of the European Union or granting Authority REA (the European Research Executive Agency). Neither the European Union nor the granting authority can be held responsible for them.

## Declaration of competing interest

The authors declare the following financial interests/personal relationships which may be considered as potential competing interests: Kerry Peek is employed by FIFA as a medical researcher, brain health. All other authors declare no competing interests.
