# Supplementary material for: Mapping the existence and content of heading coaching documents across FIFA members associations
Source: JSAMS Plus. 2026 Jul 15;8:100149. doi: 10.1016/j.jsampl.2026.100149 (PMC13383939; doi:10.1016/j.jsampl.2026.100149)
Supplement: Multimedia component 1 [file mmc1.docx]

# **Supplementary Appendices**

**Appendix 1. Method section flowchart**


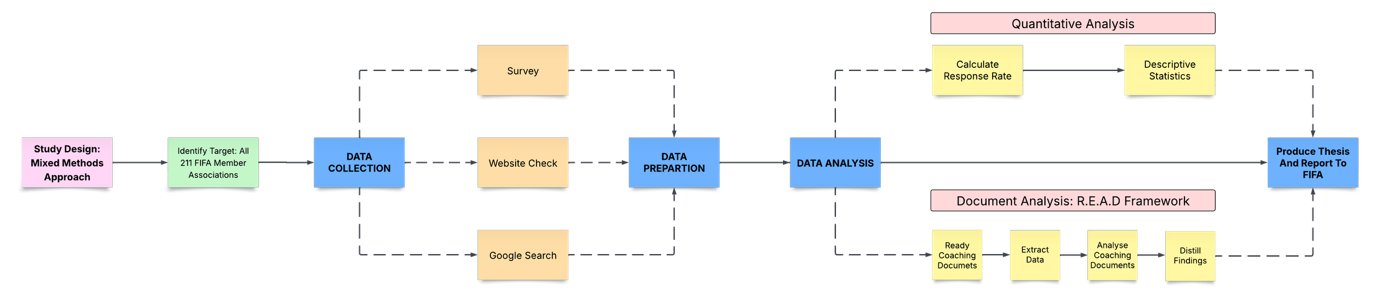


**Appendix 2. Survey response rates by confederation**

| **Confederation** | **No / No response** | **Not Sure** | **Yes** | **Total** |
| --- | --- | --- | --- | --- |
| **AFC (Asia)** | 5 | 9 | 4 | 18 |
| **CAF (Africa)** | 10 | 8 | 4 | 22 |
| **UEFA (Europe)** | 5 | 9 | 5 | 19 |
| **CONMEBOL (South America)** | 4 | 0 | 1 | 5 |
| **CONCACAF (North/Central America & Caribbean)** | 7 | 1 | 2 | 10 |
| **OFC (Oceania)** | 1 | 0 | 0 | 1 |
| **TOTAL** | 33 | 28 | 13 | 74 |

**Appendix 3. Association response rate by confederation**

| **Confederation** | **Associations Responded** | **Total Associations** | **Response Rate (%)** |
| --- | --- | --- | --- |
| **AFC (Asia)** | 18 | 47 | 38.3 |
| **CAF (Africa)** | 22 | 54 | 40.7 |
| **CONCACAF (North America/Caribbean)** | 9 | 35 | 25.7 |
| **CONMEBOL (South America)** | 5 | 10 | 50.0 |
| **OFC (Oceania)** | 1 | 11 | 9.1 |
| **UEFA (Europe)** | 16 | 55 | 29.1 |
| **Total** | 71 | 211 | 33.6 |

**Appendix 4. URL’s for publicly available Member Association coaching documents**

| Member Association | Type of Document | URL |
| --- | --- | --- |
| Uruguayan Football Association | Curriculum | <https://flacso.edu.uy/wp-content/uploads/2018/06/Guia-F%C3%BAtbol-Version-Final.pdf> |
| Football Association of Singapore | Curriculum | [https://fas.org.sg/wp-content/uploads/2024/01/National-Football-Curriculum-U13-to-U18.pdf https://fas.org.sg/wp-content/uploads/2024/01/National-Football-Curriculum-U8-to-U12.pdf](https://fas.org.sg/wp-content/uploads/2024/01/National-Football-Curriculum-U8-to-U12.pdf) |
| Philippine Football Federation | Curriculum | https://pff.org.ph/wp-content/uploads/2025/06/TAD-TR-2019.pdf https://drive.google.com/file/d/17se70RW3ffpb-uQUffK3g6p2_yHhSBti/view |
| South African Football Association | Curriculum | https://www.safa.net/wp-content/uploads/2025/04/Teaching_methodology.pdf https://www.safa.net/wp-content/uploads/2025/04/Characteristics_of_children_and_educational_approach.pdf https://www.safa.net/wp-content/uploads/2025/04/TheSAFP.pdf |
| New Zealand Football | Curriculum | <https://www.sporty.co.nz/asset/downloadasset?id=cbfee656-eca4-42d4-a8b0-8c916375f7ff> |
| Irish Football  Association | Curriculum | https://www.irishfa.com/media/28723/skills-development-programme-coaching-manual-10-12-years.pdf https://www.irishfa.com/media/28725/skills-development-programme-coaching-manual-5-6-years.pdf https://www.irishfa.com/media/28727/skills-development-programme-coaching-manual-7-9-years.pdf |
| Football Australia | Curriculum | [https://www.footballaustralia.com.au/sites/ffa/files/2017-09/FFA%20National%20Curriculum_1ma6qrmro1pyq10gzxo5rcn7ld.pdf https://www.playfootball.com.au/sites/play/files/2021-01/The%20Football%20Coaching%20Process.pdf https://playfootball.com.au/sites/play/files/2020-01/MiniRoos-Activity-Guide.pdf https://playfootball.com.au/sites/play/files/2018-08/FOOTBALL_Skill%20Acquisition%20Manual_A4_Web_Single%20Pages[1].pdf https://playfootball.com.au/sites/play/files/2021-01/The%20Football%20Coaching%20Process.pdf](https://www.footballaustralia.com.au/sites/ffa/files/2017-09/FFA%20National%20Curriculum_1ma6qrmro1pyq10gzxo5rcn7ld.pdf) |
| Football Association of Wales | Guideline + Curriculum | [Guideline available upon reasonable request https://media-faw-cymru.s3.eu-west-2.amazonaws.com/faw/20230510111722/The-FAW-National-Syllabus.pdf](https://fifa-my.sharepoint.com/personal/kerry_peek_fifa_org/_layouts/15/onedrive.aspx?ga=1&id=%2Fpersonal%2Fkerry%5Fpeek%5Ffifa%5Forg%2FDocuments%2FHeading%20guidelines%20and%20coaching%20frameworks%2FHeading%20guidelines%2FFAW%2DHeading%2DGuidelines%2DMay%2D2023%20%28Wales%29%2Epdf&parent=%2Fpersonal%2Fkerry%5Fpeek%5Ffifa%5Forg%2FDocuments%2FHeading%20guidelines%20and%20coaching%20frameworks%2FHeading%20guidelines) |
| Japan Football Association | Guideline + Curriculum | [Guideline available upon reasonable request](https://fifa-my.sharepoint.com/personal/kerry_peek_fifa_org/_layouts/15/onedrive.aspx?ga=1&id=%2Fpersonal%2Fkerry%5Fpeek%5Ffifa%5Forg%2FDocuments%2FHeading%20guidelines%20and%20coaching%20frameworks%2Fcoaching%20resources%2FJFA%28Japan%29%5Fheading%5Fguidelines%2Dtranslation%2Epdf&parent=%2Fpersonal%2Fkerry%5Fpeek%5Ffifa%5Forg%2FDocuments%2FHeading%20guidelines%20and%20coaching%20frameworks%2Fcoaching%20resources) |
| Malta Football Association | Guideline | Guideline available upon reasonable request |
| Finnish Football Association | Guideline | Guideline available upon reasonable request |
| German Football Association | Guideline | Guideline available upon reasonable request |
| Eswatini Football Association | Curriculum | Guideline available upon reasonable request |
| Scottish Football Association | Guideline | [https://www.scottishfa.co.uk/media/9832/heading-guidance-adult-football-18plus.pdf https://www.scottishfa.co.uk/media/6054/scottish-fa-heading-guidelines.pdf](https://www.scottishfa.co.uk/media/9832/heading-guidance-adult-football-18plus.pdf) |
| Norwegian Football Association | Guideline + Curriculum | [Guideline available upon reasonable request https://www.fotball.no/globalassets/samfunnsansvar-og-verdier/utenlandsk-undervisning/gfcc-part-2.pdf](https://fifa-my.sharepoint.com/personal/kerry_peek_fifa_org/_layouts/15/onedrive.aspx?ga=1&id=%2Fpersonal%2Fkerry%5Fpeek%5Ffifa%5Forg%2FDocuments%2FHeading%20guidelines%20and%20coaching%20frameworks%2FHeading%20guidelines%2FThe%20Football%20Association%20of%20Norway%5FRetningslinjer%2520for%2520heading%2520i%2520barne%2Epdf&parent=%2Fpersonal%2Fkerry%5Fpeek%5Ffifa%5Forg%2FDocuments%2FHeading%20guidelines%20and%20coaching%20frameworks%2FHeading%20guidelines) |
| US Soccer | Guideline + Curriculum | [Guideline available upon reasonable request https://cdn2.sportngin.com/attachments/document/0073/5091/Full_U.S._Soccer_Coaching_Curriculumnew.pdf](https://fifa-my.sharepoint.com/personal/kerry_peek_fifa_org/_layouts/15/onedrive.aspx?ga=1&id=%2Fpersonal%2Fkerry%5Fpeek%5Ffifa%5Forg%2FDocuments%2FHeading%20guidelines%20and%20coaching%20frameworks%2FHeading%20guidelines%2FUS%20Soccer%20Heading%20Guidelines%2Epdf&parent=%2Fpersonal%2Fkerry%5Fpeek%5Ffifa%5Forg%2FDocuments%2FHeading%20guidelines%20and%20coaching%20frameworks%2FHeading%20guidelines) |
| England Football Association | Guideline + Curriculum | [https://www.thefa.com/-/media/thefacom-new/files/rules-and-regulations/2023-24/heading-guidance/youth-heading-guidance-chart.ashx https://www.thefa.com/-/media/thefacom-new/files/rules-and-regulations/2021-22/heading-guidance/adult-amateur-heading-guidance---august-2021.ashx https://www.thefa.com/-/media/thefacom-new/files/rules-and-regulations/2021-22/heading-guidance/professional-football-heading-in-training-guidance---july-2021.ashx https://www.thefa.com/-/media/thefacom-new/files/rules-and-regulations/2021-22/heading-guidance/professional-football-heading-in-training-guidance-summary---july-2021.ashx https://www.thefa.com/-/media/thefacom-new/files/rules-and-regulations/2021-22/heading-guidance/professional-game-heading-in-training-guidance-faqs---july-2021.ashx https://www.englandfootball.com/participate/learn/brain-health/heading-in-football](applewebdata://CA45D70E-6A2A-4A6A-BE84-1478A3CC0D6B/../../../../../../../../../../Downloads/youth-heading-guidance-chart%20(3).pdf) |
| Montserrat Football Association | Guideline | [Guideline available upon reasonable request](https://fifa-my.sharepoint.com/personal/kerry_peek_fifa_org/_layouts/15/onedrive.aspx?ga=1&id=%2Fpersonal%2Fkerry%5Fpeek%5Ffifa%5Forg%2FDocuments%2FHeading%20guidelines%20and%20coaching%20frameworks%2Fcoaching%20resources%2FHeading%20Protocol%5FMontserrat%2Epdf&parent=%2Fpersonal%2Fkerry%5Fpeek%5Ffifa%5Forg%2FDocuments%2FHeading%20guidelines%20and%20coaching%20frameworks%2Fcoaching%20resources) |
| Football Association of Hong Kong | Curriculum | https://resource.hkfa.com/upload/pdf/2016/Phase%204%20Competition%20Phase.pdf https://resource.hkfa.com/upload/pdf/2016/Phase%203%20Team%20Develop%20Phase.pdf https://resource.hkfa.com/upload/pdf/2016/Phase%202%20Individual%20Skill%20Development%20Phase.pdf https://resource.hkfa.com/upload/pdf/2016/Phase%201%20Exploration%20Phase.pdf https://resource.hkfa.com/upload/Football_curriculam/Curriculum_English_Full_Version.pdf |
